# Arthritogenic Alphaviruses: A Worldwide Emerging Threat?

**DOI:** 10.3390/microorganisms7050133

**Published:** 2019-05-14

**Authors:** Laura I. Levi, Marco Vignuzzi

**Affiliations:** 1 Populations Virales et Pathogenèse, Institut Pasteur, CNRS UMR 3569, 75015 Paris, France; laura.levi@pasteur.fr; 2 Ecole doctorale BioSPC, Université Paris Diderot, Sorbonne Paris Cité, 75013 Paris, France

**Keywords:** alphaviruses, chikungunya virus, Mayaro virus, O’nyong’nyong virus, Ross River virus

## Abstract

Arthritogenic alphaviruses are responsible for a dengue-like syndrome associated with severe debilitating polyarthralgia that can persist for months or years and impact life quality. Chikungunya virus is the most well-known member of this family since it was responsible for two worldwide epidemics with millions of cases in the last 15 years. However, other arthritogenic alphaviruses that are as of yet restrained to specific territories are the cause of neglected tropical diseases: O’nyong’nyong virus in Sub-Saharan Africa, Mayaro virus in Latin America, and Ross River virus in Australia and the Pacific island countries and territories. This review evaluates their emerging potential in light of the current knowledge for each of them and in comparison to chikungunya virus.

## 1. Introduction

Alphaviruses are a genus of enveloped positive-stranded RNA viruses belonging to the *Togaviridae* family. As arboviruses, they circulate between an invertebrate vector (mosquitoes) and a vertebrate host. They are distributed worldwide and are separated into two groups: the New World alphaviruses responsible for disease with neurological involvement and the Old World alphaviruses (or Semliki forest virus group) that have a rheumatic tropism. The latter, also called arthritogenic alphaviruses, notably include chikungunya virus (CHIKV), now widely distributed, O’nyong’nyong virus (ONNV) located in Sub-Saharan Africa, Mayaro virus (MAYV) in Latin America, and Ross River virus (RRV) in Australia and the Pacific island countries and territories [1] (Table 1).

CHIKV emergence in the Indian Ocean islands in 2005–2006 [2,3] and throughout the Caribbean states and the Americas in 2013–2014 [4] has raised awareness of the threat that other alphaviruses represent. While ONNV, MAYV, and RRV are currently responsible for smaller, local epidemics, the risk of dissemination worldwide is real, promoted by international trade and travel, globalization, and climate change.

In this work, we review the current knowledge on CHIKV and compare it to these three less-described alphaviruses (ONNV, MAYV, and RRV) circulating in three distinct continents, to try to understand their emergence potential in the near future.

## 2. Historical Description and Main Epidemics

### 2.1. Chikungunya Virus

Robinson first described chikungunya fever in 1955, after an epidemic of dengue-like fever with extremely severe joint pain in Southern Tanganyika (now Tanzania) in 1952–1953 [7]. Its name comes from the Kimakonde language meaning “that which bends up” describing this particular clinical manifestation.

Three main chikungunya virus genotypes, named after their original geographical distribution, are described: the West African, the East-Central-Southern African (ECSA, including Indian Ocean lineage), and the Asian genotypes (that include the Caribbean strains) [8,9]. Before 2000, chikungunya virus had been circulating mainly in Sub-Saharan Africa (Zimbabwe [10,11], Nigeria [12], Angola [13], South Africa [14], Democratic Republic of Congo [15,16], Senegal [17,18]) and South-East Asia (India [19,20,21,22], Sri-Lanka [21], Vietnam [23], Thailand [24], Malaysia [25], Indonesia [26,27]) where numerous local outbreaks have been reported. Since then, two worldwide epidemics occurred that pushed chikungunya virus into the spotlight.

The first epidemic, caused by the Indian Ocean lineage, started in coastal Kenya in august 2004 with an attack rate of over 50% [2] and rapidly disseminated to the Comoros islands in December 2004–March 2005 [28], and from there to Mayotte, Mauritius, La Reunion, and the Seychelles [29], infecting hundreds of thousands of people at the end of 2005 through the beginning of 2006. Because La Reunion Island is a French territory with a developed public health infrastructure and surveillance system, the epidemic gathered more global attention after making the headlines. It also disseminated through India and South-East Asia, and into southern Europe because of air travel, causing altogether approximately six million cases in more than 40 countries [2,3].

In December 2013, the Asian genotype was responsible for the second major outbreak that started in Saint Martin Island in the Caribbean Sea [30,31]. From there, it disseminated throughout the American continent, causing more than two million cases in 50 countries [4].

A particular and recent concern about CHIKV is its potential to cause outbreaks in temperate regions where the Asian tiger mosquito, *Aedes albopictus*, has recently established, enabling local epidemics, such as in Italy in 2007 [32] and 2017 [33,34,35] and in France in 2010 [36] and 2017 [37].

### 2.2. O’nyong’nyong Virus

Although genetically distinct from CHIKV, ONNV is its most closely related relative [8]. Similar to CHIKV, it can cause large-scale epidemics in Africa, but infection by ONNV has never been reported outside of this continent. The first described ONNV outbreak took place during the first half of 1959 in north-western Uganda (Gulu), before it crossed the border to Belgian Congo and Sudan, causing close to 750,000 cases [38]. This epidemic lasted until 1962, spreading from eastern to western Africa and permitting the first isolation of the virus in human serum [39]. In 1984–1985, it caused another outbreak in Ivory Coast [40], and although it was then called Igbo-Ora virus, it is now clear that it was in fact a lineage of ONNV [8,41]. A third epidemic took place in southern Uganda in 1996–1997 with 40–80% of the population infected [42,43]. More recently (2003), an outbreak in a Liberian refugee camp was reported in Ivory Coast [44].

### 2.3. Mayaro Virus

MAYV was first described in Trinidad where it caused mild to moderate short febrile illness in five humans in 1954 [45]. In 1959, it caused an epidemic among colonists in the Bolivian rain forest under the name of Uruma virus, which has since been proven to be MAYV [46]. Since then, it has been responsible for several small outbreaks or sporadic cases in rural areas around tropical forests in South and Central America (Brazil [47,48,49,50,51], Bolivia [52], Ecuador [53], Venezuela [54,55], Peru [56,57], French Guyana [58], Suriname [59], Mexico [60], Haiti [61]).

### 2.4. Ross River Virus

Epidemic polyarthritis has been described since 1886 in rural Australia [62,63]. The virus responsible for this was first isolated in 1959 from infected mosquitoes along the Ross River in Queensland, Australia [64]. Because patients suffering from epidemic polyarthritis had antibodies against RRV, it was thought to be the agent responsible for the disease [65,66]. In 1971, it was first isolated from an aboriginal boy in Australia [67], confirming this hypothesis. Since then, frequent RRV outbreaks have been reported in Australia [68,69,70] with close to 5000 cases/year [71]. RRV also circulates in the Pacific island countries and territories [72] since it was introduced in 1979–1980, causing an epidemic with over 500,000 cases and spectacular attack rates in Fiji (90%), the Cook Islands (69%), American Samoa (44%), and New Caledonia (33%) [73,74,75].

## 3. Sylvatic Versus Urban Cycle

### 3.1. Chikungunya Virus

Between outbreaks, CHIKV circulates in a sylvatic cycle where non-human primates are the main host [76,77,78,79,80], and sylvatic *Aedes* mosquitoes (such as *Aedes africanus* [78], *Aedes furcifer-taylori*, *Ae. luteocephalus*, *Ae. Dalzieli* [18], etc.) are the main vector. Even though these mosquitoes are not particularly anthropophilic, sporadic human cases can originate from that cycle if a human is bitten by an infected mosquito in a rural area. While the western African lineage has never switched to an urban cycle, both the Asian and the ECSA lineages are able to circulate in urban cycles between humans (that become the amplifying host) [81] and anthropophilic urban mosquitoes (*Aedes aegypti* and *Aedes albopictus*) [7,38,82]. Interestingly, while *Aedes aegypti* is usually the main vector of CHIKV in African urban cycles, *Aedes albopictus* (widely present on La Reunion Island in the Indian Ocean) was the vector responsible for the transmission during the epidemic that bore its name. Indeed, a single mutation, A226V in the E1 glycoprotein, favored the infection and dissemination of the virus in the new *Aedes albopictus* vector [83,84,85]. Interestingly, this same A226V mutation appeared independently in India, Sri Lanka, Cameroon, and Gabon in settings where *Aedes albopictus* was suspected to be the vector of the disease. This convergent evolution illustrates the ease with which the virus evolves to improve its transmissibility [4].

### 3.2. O’nyong’nyong Virus

Mainly circulating in an enzootic cycle, the vertebrate host of ONNV is not clearly defined. Nevertheless, forest buffalos, mandrills, and duikers were positive for ONNV in a serosurvey in the Congo basin [86]. *Anopheles funestus* and more rarely *Anopheles gambiae* are the main vectors [38,87,88,89]. Experimentally, ONNV can infect *Aedes aegypti* and disseminate to their salivary glands [90], but *Aedes* mosquitoes have not yet been demonstrated to be carriers in the wild. More likely, as for CHIKV, it switches to an urban cycle where humans are the amplifying host during bigger outbreaks.

### 3.3. Mayaro Virus

MAYV is circulating in an enzootic cycle between non-human primates [58,91,92,93,94] (or more rarely, small mammals [92] or birds [95]) and forest mosquitoes including *Haemagogus* sp. [49,91], *Mansonia* sp. [96], and *Psophora* sp. [5,6]. Nonetheless, two studies found pools of *Aedes aegypti* positive for MAYV [6,97], and experimental work confirms that they might be efficient vectors for the disease, as well as *Aedes albopictus* [98,99,100,101,102]. This raises the possibility of MAYV circulating in an urban setting. Furthermore, in an experimental setting, four *Anopheles* species (two North American species, one African, and one Southeast Asian) were able to get infected and to transmit MAYV, adding other potent MAYV vectors distributed worldwide to the list [101].

### 3.4. Ross River Virus

While twenty-one vertebrate hosts have been described for RRV [103], marsupials are considered to be the most important reservoir [104,105,106], even though proof is scarce [107], and some locations such as the islands of Samoa have circulation of RRV without the presence of marsupials [108]. Horses or cattle also play a role [105,109], and humans are the main host during outbreaks [106]. Different species of mosquitoes seem to play a role in the RRV cycle. *Aedes vigilax* are the most common ones in coastal and subcoastal areas and *Culex annulirostris* in both rural and urban settings, but *Aedes notoscriptus*, *Aedes funereus*, *Aedes camptorhynchus*, and *Ochlerotatus camptorhynchus* are also incriminated [69,70,110]. Experimentally, *Aedes aegypti* and *albopictus* are efficient vectors [111,112,113,114], but it has never been reported to happen in the wild.

## 4. Clinical Description

**Figure 1:**
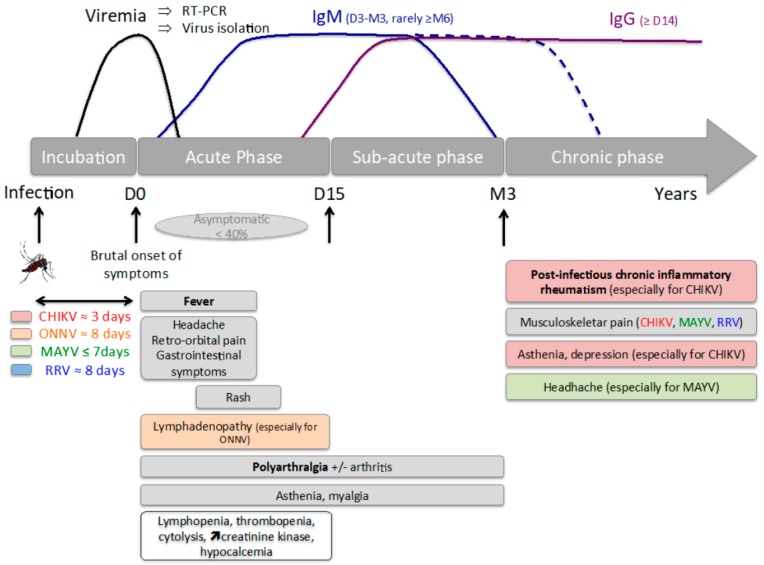
Timeline after arthritogenic alphavirus infection. After infection by a bite from an infected mosquito, an incubation period of 3–8 days takes place. Viremia starts before the onset of symptoms and lasts only a few days, leaving a very short window for direct diagnosis by RT-PCR or virus isolation. If the infection is symptomatic (>60% of cases), the patient presents with brutal onset of fever associated with polyarthralgia and other general symptoms, and after a few days, a delayed rash often appears. Lymphadenopathy is particularly frequent in ONNV infection. General blood tests during the acute phase can show lymphopenia, thrombopenia, hypocalcemia and mild rhabdomyolysis (with cytolysis and increased creatinine kinase). Polyarthralgia with or without arthritis and asthenia might be prolonged during the sub-acute phase (2 weeks to 3 months) or as chronic symptoms, sometimes as post-infectious chronic inflammatory rheumatism mimicking rheumatoid arthritis. Indirect diagnosis by serology requires two blood samples separated by at least 12 days to show appearance and/or disappearance of IgM (that may stay positive for over 6 months) and appearance of IgG.

**Figure 2:**
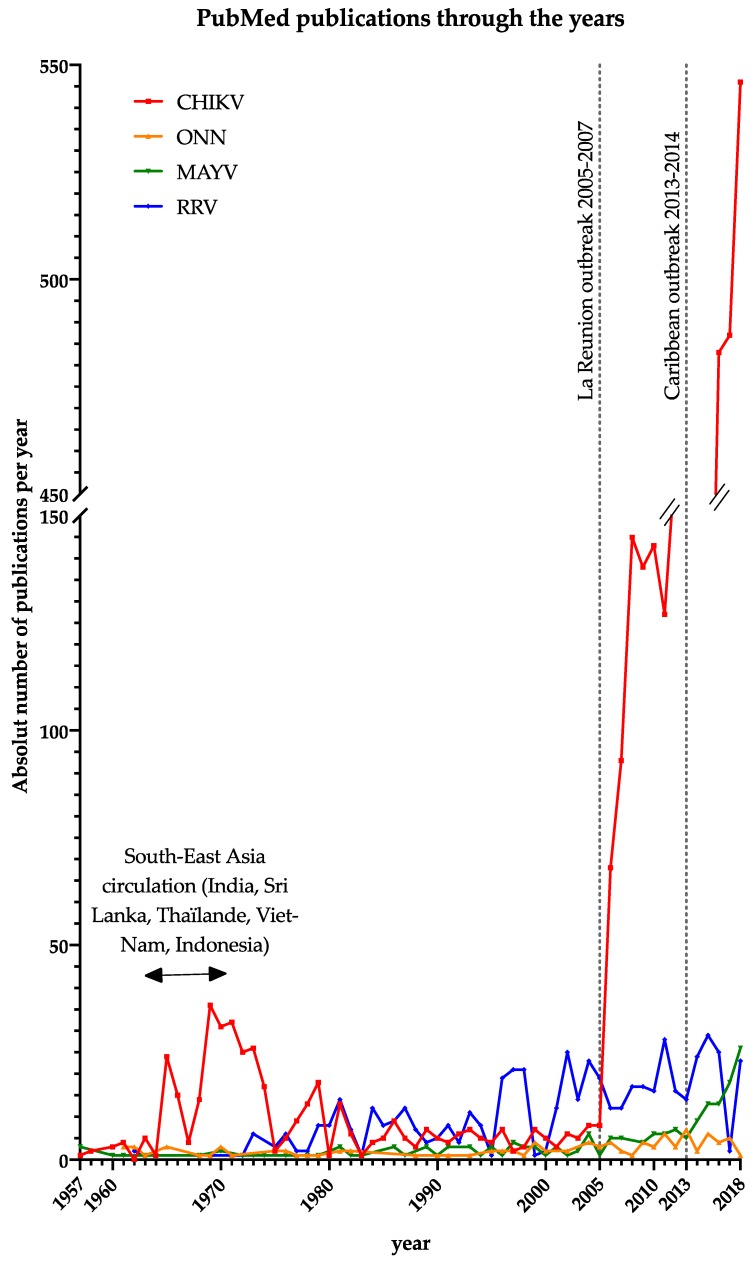
Number of publications for each virus reported in PubMed through the years. CHIKV publications were rare before 2006 (under 10 per year), except during the South-East Asia epidemic in the late 1960s, when it reached around 20 publications per year. Since the La Reunion outbreak, and followed by the Caribbean epidemic, attention has increased tremendously, and publications now number around 500 per year. While Ross River virus has 10–20 publications a year since 1979, MAYV and ONNV had less than 10 publications a year until 2015, and only MAYV has slightly exceeded this number since then.

Contrary to what has been reported for most flaviviruses (for which asymptomatic cases can reach up to 94% [115,116]), most alphavirus infections are symptomatic with rates ranging from 72–97% for CHIKV [30,117,118,119,120] (only one study reporting an unusually low symptomatic infection rate of 18% [121]), 64–86% for ONN [42,122,123,124], 64–80% for MAYV [48,56], and 25–76% for RRV [73,125,126,127]. Rheumatic disease is the main clinical manifestation compared to other arboviral infections that are responsible for a flu-like syndrome. They all follow roughly the same timeline (Figure 1) and share the same major symptoms (Table 2). The severity of these infections, however, is difficult to compare between viruses because of considerable variability in absolute numbers and methods of clinical study (Figure 2). 

### 4.1. Acute Phase

Following a 2.5-day incubation period (range of 2–12 days) [7,162], the acute phase of CHIKV infection is characterized by a brutal onset of symptoms typically associated with fever, very severe polyarthralgia (“frightening in its severity” as first described by Robinson [7]) with possible synovitis, and a delayed rash that is often pruriginous while rarely bullous [128,163,164]. Less well-known, but commonly associated manifestations include headaches, gastrointestinal symptoms, and more rarely conjunctival hyperemia or lymphadenopathy. While in the earliest descriptions of the disease the hemorrhagic, severe or lethal cases were questioned because of co-circulation with dengue fever [19,129,165,166], it is now clearly established that CHIKV infection can carry potentially lethal neurologic and cardiologic involvement [150,152,153] or be directly or indirectly linked to death, particularly in older patients with numerous comorbidities [128,150,156,167] or in neonates [156,157]. Overall, the global mortality rate is estimated at 0.01–0.1% [32,81,119,156].

The ONNV fever acute phase follows an eight-day or more incubation period [122]. It is characterized by the triad of fever, polyarthralgia, and lymphadenopathy. This last sign is more specific to ONNV infection and is mostly located in the cervical region. Skin rash is associated a few days later and is often itchy. Other reported complications include headaches, gastrointestinal symptoms, and eye involvement [44,122,141]. Bleeding gums and epistaxis have been reported for one patient each [141]. To our knowledge, no mortality linked to ONNV infection has been described.

After less than a week of incubation [47], MAYV infection causes brutal fever associated with arthromyalgia (sometimes with swollen joints). Headaches, retro-orbital pain, and gastrointestinal symptoms are frequent [47,49,50,51,56,142,143,144,145]. This acute phase usually lasts two to five days [49]. Three independent studies reported benign hemorrhagic signs in a total of seven patients (bleeding gums, epistaxis, petechiae) [56,60,142]. Only one patient with heavy comorbidities was reported to have died following MAYV infection, without certainty about the cause of death [60].

From traveler data, the incubation time of RRV infection is estimated to be 7–9 days [168] (ranging between three and 21 days [75,169]). Fever polyarthralgia and rash are, as is the case for the other rheumatic alphaviruses, typical, occurring during the acute phase [125,146,147,148,149]. If only nausea is reported in the main cohorts or retrospective studies, gastrointestinal symptoms are often mentioned in case reports [72,170]. Severe forms of infection include glomerulonephritis [158] and encephalitis [159,160,161], but to our knowledge, no lethality has been reported.

### 4.2. Chronic Phase

With over 40 clinical studies to date, the chronic joint pain (i.e., persisting after 12 weeks of onset of symptoms) is very well described and represents the major burden of CHIKV infection because it can be responsible for very heavy and long term incapacity [130]. A recent meta-analysis estimated that 43% of patients after three months and 21% after one year still experience symptoms linked to CHIKV. This rate seems to depend on which CHIKV strain is involved [171]. In a recent prospective cohort of La Reunion Island patients [172], chronic joint pain concerned up to 83% of patients at 32 months and was accompanied by asthenia and depression [173]. As the virus has never been isolated from chronic swollen joints, it is thought that CHIKV acute infection might trigger host autoimmunity [172,174,175]. However, it is important to differentiate chronic inflammatory rheumatism causing erosive arthritis [131] and for which the differential diagnosis is rheumatoid arthritis, from musculoskeletal disorder. Indeed, whereas the latter is only treated with pain killers possibly associated with steroids, the former requires a heavier treatment including methotrexate [176].

At the opposite end of the spectrum, accounts of ONNV chronic manifestations are scarce. While Shore et al. mentioned joint pain associated with residual weakness and depression in some patients in 1965 [122], other studies either report “no recurrent fever case” [141] or do not mention chronicity [44].

For MAYV, recurrent arthralgia has been reported in one patient [177], and some case reports have mentioned arthralgia lasting over 5–12 months after MAYV infection [178,179,180,181]. In 2013, one Peruvian prospective study of 13 patients confirmed these data and evaluated chronic arthralgia and headache to be present in 57% and 31% of patients 12 months after infection [143].

As for CHIKV, there is great heterogeneity between studies concerning chronic joint pain after RRV infection. Two studies reported them in 52.5% of patients at one year and 57% of patients at three years [146,147]. Two other studies reported progressive resolution of symptoms in 3–6 months [149] or a prevalence of chronic arthralgia in only 2% of patients with no other conditions than RRV infection [148]. This last study, apart from insisting on the difficulty of differentiating post-infectious chronic arthralgia from differential diagnosis, also implied a higher prevalence of chronic joint pain in the elderly, that also present with more comorbidities (even though this was not discussed in the paper). Although there is no human data available, in vitro and in vivo data suggest that post-RRV infection arthritis may be erosive [182,183].

## 5. Laboratory Features

### 5.1. Standard Blood Test

For CHIKV, standard biological tests usually show lymphopenia, more rarely associated with a moderate thrombocytopenia. Hypocalcemia is detected in more than half of the cases, and a mild rhabdomyolysis with moderately elevated creatinine kinase and transaminases (ASAT > ALAT) might be observed [128,132,133,134]. Very few data are available for the other three viruses, but leucopenia and thrombopenia have also been described for MAYV.

### 5.2. Positive Diagnosis

Viremia usually rises a few days before onset of symptoms. RT-PCR or virus isolation permit direct diagnosis; however, most of the time, RT-PCR can no longer detect virus seven days after onset of symptoms for CHIKV [32,184] (even though one patient had a positive RT-PCR 17 days after [185]) and for MAYV [186], and virus isolation is positive for an even shorter time [187]. This diagnostic tool is particularly useful because test specificity is very good between, and within, viral families, as specific assays exist for each virus [186,188,189,190].

Indirect diagnosis is achieved by serological tests to detect IgM and IgG. The antibodies arise later than viremia, but are long lasting in comparison. Indeed, as it has been shown for CHIKV, IgM can arise as soon as two days after onset of symptoms and is usually positive after a week [184,191]. IgM levels usually wane after three months [191], but in some cases can persist more than six months [128,192]. IgG usually appears after 7–14 days of the onset of illness and is long lasting [184,191]. Whatever the commercial or in-house serological test used (most often by enzymatic immune assay or hemagglutination inhibition), two samples per patient, taken at least 10 days apart, are needed to show the appearance of IgM and/or IgG or disappearance of IgM.

The main pitfall of serological methods is the cross-reactivity among alphaviruses that complicates identifying which of the arthritogenic alphaviruses is involved, especially in regions where they co-circulate. For this reason, the plaque-reduction neutralizing test (PRNT) remains a gold standard for diagnostics [193,194].

## 6. Treatment

### 6.1. Prevention

Because an efficient curative treatment does not exist, prevention measures are crucial to avoiding or limiting epidemics caused by these arboviruses. These include general measures to avoid mosquito bites and vaccination.

#### 6.1.1. Public Health Measures: Surveillance and Vector Control

Public health response measures are essential to prevent and control any alphavirus outbreak. During disease-free periods, ongoing mosquito surveillance (e.g., population densities, distribution) has shown its effectiveness for predicting RRV epidemics in Australia [195]. Early detection of outbreaks is also favored by notification of cases, something that is mandatory for RRV in Australia and CHIKV in many other countries, leading to the early detection and increased surveillance of outbreaks [71,196,197,198,199]. As soon as detection occurs, intervention in mosquito habitats, using a combination of larvicides, adulticides, and removal of breeding sites, may be the best strategy [198,200,201,202]. Geovisualization tools might also help locate the source of infection to better target it [203]. Public outreach to educate individuals on how to avoid mosquito bites (e.g., protective clothing, mosquito repellents, and impregnated bed nets) is also common, but its effectiveness is still not clearly demonstrated [200].

#### 6.1.2. Vaccination

Because CHIKV infection produces a life-lasting protection against a second infection and due to the limited antigenic variability of the virus, one might think that a CHIKV vaccine should be relatively easy to produce, or at least easier so than a dengue virus vaccine [204]. Nevertheless, there are still no licensed CHIKV vaccines available, although many candidates have been tested including live-attenuated vaccines [205,206], recombinant virus vaccines (measles virus [207,208,209], vesicular stomatitis virus [210,211]), inactivated vaccines [212,213], virus-like particles (VLPs) [214,215,216], DNA- [217,218] or mRNA-based vaccines [219], and subunit formulations of CHIKV [220].

Two candidates are currently in Phase II clinical trials. The first one is a recombinant measles virus encoding the CHIKV structural proteins. The first results of the Phase II trial are very encouraging with all vaccines fully immunized after two injections (regardless of the individual’s immunological status against measles virus) and excellent safety and tolerability [209]. The second candidate is a VLP vaccine containing CHIKV structural proteins that is immunogenic and protective against a challenge infection in mice and rhesus macaques [214]. A Phase I clinical trial confirmed immunogenicity in humans after two injections and the absence of serious adverse events [221,222]; a Phase II trial is currently underway.

While it still needs to be unequivocally demonstrated, a number of studies suggest that protection against one strain of CHIKV protects against all others [223,224,225,226]. Interestingly, Partidos et al. showed that passive monoclonal antibodies after immunization of mice with a CHIKV vaccine protect against ONNV infection (the closest related alphavirus) in AG129 mice [227]. Because there are no vaccine candidates for ONN, a broad-spectrum CHIKV vaccine is of particular interest; that should also be evaluated for MAYV and RRV.

Three vaccine candidates have been tested for MAYV. The first one was a formalin-inactivated vaccine with moderate efficiency in mice [228]. More recently, a live attenuated vaccine and a DNA vaccine showed good immunogenicity and protection after a lethal challenge in mice [229,230].

Since RRV has been a public health issue for some time now in Australia, vaccine research there has led to several candidates in preclinical studies [231,232,233,234], and formalin-inactivated vaccines have successfully passed Phase I, II, and III clinical trials [235,236]. However, this vaccine is not commercialized, and no RRV vaccine is currently available, highlighting the importance of public/private partnerships to invest in vaccine licensure [237].

### 6.2. Symptomatic and Curative Treatments

Symptomatic treatment with antalgics, such as paracetamol, and steroidal or non-steroidal anti-inflammatory drugs remains the main driver of action available [148,238,239]. As discussed above, in case of chronic inflammatory rheumatism, methotrexate treatment might be necessary [176].

Several studies have searched for efficient antiviral molecules that have a broad-spectrum activity inhibiting all arthritogenic alphaviruses [240,241,242,243,244,245,246]; however, despite a long list of antivirals efficient in vitro or in vivo, no treatment is currently available [247]. Indeed, all clinical trials conducted until now have either failed, such as for chloroquine [248,249], or have been inconclusive because of small test groups, such as for ribavirin [250]. Pentosan polysulfate has shown efficiency against cartilage damage in mice both with CHIKV and RRV, and a Phase II trial is now ongoing [251].

Another approach is to use hyper-immune intravenous anti-CHIKV immunoglobulins to prevent CHIKV infection in neonates, who often develop very severe forms of the disease. Indeed, after a conclusive study in neonate mice [252], a Phase II trial is currently in progress [253].

## 7. Discussion

With two worldwide epidemics causing close to 10 million cases all together during the 21^st^ Century, chikungunya virus is undoubtedly a re-emerged disease threatening both tropical and temperate regions [2,3,4]. While it was a neglected tropical disease until the early 2000s, it has since gathered considerable interest as the number of publications through the years illustrates (Figure 2). In contrast, ONN, MAYV, and RRV are still understudied, even though specialists consider them as a Sword of Damocles. Indeed, some reviews raise the question of a possible emergence in the near future [186,254,255,256,257], especially for ONNV, which is known to have caused a large African outbreak with a hundred thousand cases in 1959–1962 [38].

One of the prerequisites to imagining a worldwide outbreak of any of these alphaviruses is a wide distribution of their mosquito vector. Indeed, no circulation of the disease would occur in a region free from competent mosquitoes. Interestingly, none of these four alphaviruses shares the same vector (Table 1), and they are currently more geographically restrained than the CHIKV vector: *Anopheles* sp. for ONNV in Africa and the Middle East, *Haemagogus* sp., *Mansonia* sp., and *Psophora* sp. for MAYV in America. and *Aedes vigilax* and *Culex annulirostris* for RRV in Oceania and South-East Asia [258]. However, globalization, travel, migration, trade, or climatic change might be leading forces of the redistribution of these mosquitoes. Indeed, climate and weather patterns are important factors in the occurrence of epidemics, which have become the focus of many modeling studies. For example, natural climatic and weather trends influenced RRV’s pattern of infection, especially hydrological features [125,259] and temperatures [260,261,262]; and a modeling study predicts that global warming might lead to the presence of MAYV across all of the Brazilian territory [263].

Furthermore, *Aedes aegypti* and/or *albopictus* have been proven to be competent vectors in experimental settings for ONNV, MAYV, and RRV [6,90,97,98,99,100,102,111,112,113,114]. While this does not mean it is currently happening in the wild, these observations are a warning of what could occur in the future, particular if vector populations expand and overlap and further adaptation to new vectors occurs. Public health services should keep this on their radar, especially because alphaviruses are single-stranded RNA viruses that are known to evolve rapidly [264]. One point mutation or one recombination event might favor infection of *Aedes aegypti* and/or *albopictus* compared to the original vector and trigger a worldwide epidemic, as has happened for CHIKV (during the La Reunion epidemic with the A226V mutation [83,84,85] and during the American outbreak with a duplication of the 3’UTR [265]). Because these vectors are widely distributed anthropophilic mosquitoes, the risk of initiating an urban cycle could be high, and further enabled by high viremia in humans.

On the other hand, the real risk and the severity of an outbreak are difficult to anticipate because little is known about ONNV, MAYV, and RRV. While a surveillance system has been well established in Australia for RRV (as it is a main health problem in a developed country), under-surveillance of ONNV and MAYV is an issue, as well as the low number of clinical studies. Thus, many open questions remain concerning possible under-the-radar outbreaks, the true incidence of the diseases, or their clinical manifestations: What is the real risk of chronicity? Can severe cases occur and which types? Can these infections be directly or indirectly lethal? Since CHIKV is more of a global concern, fundamental knowledge on alphaviruses in general is expanding, and many of these publications also tested their hypothesis on the related ONNV, MAYV, or RRV. As an example, recent findings on the still obscure entry pathway of alphaviruses identified Mxra8 as an entry mediator, not only for CHIKV, but also for ONNV, MAYV, and RRV [266].

Another potential pitfall, if an epidemic were to happen, is the speed of identifying which virus is responsible. Indeed, despite some virus-specific trends such as lymphadenopathy for ONNV, arthritogenic alphaviruses share very similar clinical manifestations that are also common to other arboviruses or other infectious diseases. Because they co-circulate in many geographical areas, an etiological diagnosis without biological testing seems impossible. Moreover, many cases of arbovirus co-infections have been reported, adding a layer of complexity to this problem [19,129,165,166,267,268,269]. As a further complication, these viruses currently circulate in remote rural areas with low access to health care facilities, including laboratories. Fortunately, many tests are now better adapted to the field [270,271,272], particularly for indirect diagnosis, but also with sequencing technology, such as MinION [273]. Regardless, cross-reactivity remains a main limitation in biological diagnostics, especially for immunological tests [274,275,276]. Hence, the closer alphaviruses are phylogenetically, the more cross-reactivity exists [194]. In response, considerable effort is made on developing technologies to avoid cross-reactivity both in direct [271,277] and indirect diagnosis [194,278].

Despite the problems stemming from cross-reactivity, it could be a major help in vaccine design, allowing broad-spectrum protection across all arthritogenic alphaviruses. Indeed, broad neutralizing antibodies against CHIKV can neutralize MAYV and ONNV by inhibiting entry and egress [227,279]. While no licensed vaccine exists for any of these viruses, several candidates have gone or are currently going through clinical trials for CHIKV and RRV, and it is reasonable to expect a CHIKV vaccine in the years to come. The immunity triggered by this vaccine would then need to be evaluated on other alphaviruses to validate its potent broad-spectrum activity. Strengthening the collective commitment of national and international actors and public/private partnerships will be necessary to develop and license vaccines [237] and to avoid what had happened for RRV: an efficient and safe vaccine that went through Phase I, II, and III clinical trials [235,236], yet is still not licensed.

In conclusion, enriched by lessons learned from recent CHIKV outbreaks, it is undeniable that ONN, MAYV, or RRV represent emerging threats favored by climatic changes, globalization, travel, migration, and trade. Surveillance is key for detecting any unusual activity of these diseases, so that preventive measures can be taken as soon as possible to tackle a starting epidemic. Hopefully, the availability of a CHIKV vaccine might enable a broad-spectrum activity on all arthritogenic viruses that would be key for future prevention.

## Figures and Tables

**Table 1:** Arthritogenic alphavirus general characteristics. CHIKV, chikungunya virus; ONNV, O’nyong’nyong virus; MAYV, Mayaro virus; RRV, Ross River virus.

	CHIKV	ONNV	MAYV	RRV
**Other Name**		Uruma virus	Igbo-Ora virus	Epidemic polyarthritis
**Location of circulation**	Endemic: Africa, Asia (South-East and India), Pacific Islands, Central and South America. Sporadically: Mediterranean Europe, USA	Sub-Saharan Africa	Central and South America	Australia, South Pacific Island, Papua New Guinea
**Cycle**	Enzootic cycle and urban cycle during epidemic	Enzootic cycle and urban cycle during epidemic	Enzootic cycle	Enzootic cycle and urban cycle during epidemic
**Vertebrate host**	Non-human primate	Mostly unknown (possibly forest buffalo, monkeys, duikers)	Non-human primate	Marsupials mainly, horses, cattle
**Invertebrate vector**	*Aedes* sp. (enzootic cycle: *africanus*, *furcifer-taylori*, *luteocephalus*, *dalzieli;* urban cycle: *aegypti* and *albopictus*)	*Anopheles funestus* and more rarely *Anopheles gambiae*	*Haemagogus* sp., *Mansonia* sp., and *Psophora* sp. [5,6]	*Aedes vigilax* and *Culex annulirostris* mainly, *Aedes notoscriptus*, *Aedes funereus*, *Aedes camptorhynchus*, *Ochlerotatus camptorhynchus*

**Table 2:** Arthritogenic alphavirus acute infection symptoms, range of frequency reported in the listed studies.

	CHIKV Median	Min	Max	ONNV Median	Min	Max	MAYV Median	Min	Max	RRV Median	Min	Max
**% of symptomatic cases**	18–97 [30,117,118,119,121]			64–86 [42,122,123,124]			64–80 [48,56]			25–76 [73,125,126,127]		
**Incubation time**	2,5 days (2–12)			Approximately 8 days			<7 days			8 days (3–21)		
**Symptoms**												
**Arthralgia**	95	69	100	79	58	100	86	50	100	94.35	83	98
**Joint Stiffness**	93	93	93							84.9	78.8	89
**Fever**	94	83	100	90	72	100	100	100	100	54.4	49	60
**Asthenia**	77.5	49	91				87	42	100	92.3	91.3	93.3
**Anorexia**	70	30	86				48.5	22	75			
**Myalgia**	59	24	85	71	71	71	78.5	49	100	61.25	45	66.7
**Headache**	57.5	8	80	84.5	74	95	87	57	100	51	50.9	60
**Gastrointestinal Symptom**	55	10	63									
**Post Orbital Pain**	49.5	14	85	60	60	60	60	39	100			
**Skin Rash**	49	14	68	77	65	84	50	24	93	58.25	57	59.5
**Nausea**	44	26	69				42.5	18	69	25.3	24.6	26
**Pruritus**	30.5	14	50	87	87	87	33	20	40			
**Swollen Joints**	40	26	100				58	23	93	52.15	38	64.7
**Vomiting**	35	19	43				17.5	4	100			
**Diarrhea**	22.5	4	38				9	5	60			
**Conjunctival Hyperemia**	18.5	3	33	51	51	51						
**Abdominal Pain**	17	13	32				50	14	80			
**Lymphadenopathy**	9	3	100	46	46	46	17	13	53	10	10	10
**Number of study/total number of patients [references]**	18/4502 [21,25,32,117,128,129,130,131,132,133,134,135,136,137,138,139,140]			3/891 [44,122,141]			9/179 [47,49,50,51,56,142,143,144,145]			5/626 [125,146,147,148,149]		
**Severe forms**	Meningo-encephalopathy [150,151], Guillain-Barré syndrome [152], acute optic neuropathy [153], Myo-pericarditis [150,154,155], shock [150], neonatal infection [156,157]			unknown			unknown			Glomerulonephritis [158], encephalitis [159,160,161]		
